# Serological and molecular diagnosis of hepatitis B virus

**DOI:** 10.1186/1471-2334-12-S1-P31

**Published:** 2012-05-04

**Authors:** S Durgadevi, Rahul Dhodapkar, Subash Chandra Parija

**Affiliations:** 1 Department of Microbiology, Jawaharlal Institute of Postgraduate Medical Education and Research (JIPMER), Puducherry, India

## Background

Hepatitis B is a potentially life threatening liver infection which leads to millions of deaths annually. Serological and molecular assays for hepatitis B virus are the major diagnostic tools. Dried blood spot (DBS), a minimally invasive procedure, is an alternative to serum and can be used for field based studies for molecular detection. The present aim of this study is to diagnose HBV infection by combination of serological and molecular methods from serum and dried blood spot.

## Methods

Blood was collected from suspected cases of liver diseases attending JIPMER Hospital, during September 2010 to October 2011. The study group is divided into two each having 30 cases of hepatitis B surface marker positive and negative profile respectively. Samples were analyzed for complete serological tests (surface and core antigen) and PCR were performed on serum samples and DBS.

## Results

Out of 30 HBsAg positive cases screened by ELISA, 22 samples were found positive of HBV DNA by PCR method from serum, which includes 2 samples with only surface (HBsAg) and antibody to core antigen (IgM anti HBc) positive. All these 22 positive cases were also been detected from DBS after storing the sample at 25°C for 4 and 7 days.

## Conclusion

It is important to detect both serological and molecular markers to diagnose hepatitis B for appropriate management of disease Since dried blood spot yields comparable results to serum in detecting HBV DNA it can be used as convenient method of collecting samples than venous blood, particularly in resource limited settings.

